# Improving patient self-description in Chinese online consultation using contextual prompts

**DOI:** 10.1186/s12911-022-01909-3

**Published:** 2022-06-27

**Authors:** Xuedong Li, Dezhong Peng, Yue Wang

**Affiliations:** 1 College of Computer Science, Sichuan University, Chengdu, China; 2 School of Information and Library Science, University of North Carolina at Chapel Hill, Chapel Hill, NC USA

**Keywords:** Online health care consultation, Self-description, Machine learning, Contextual prompts

## Abstract

**Background:**

Online health care consultation has been widely adopted to supplement traditional face-to-face patient-doctor interactions. Patients benefit from this new modality of consultation because it allows for time flexibility by eliminating the distance barrier. However, unlike the traditional face-to-face approach, the success of online consultation heavily relies on the accuracy of patient-reported conditions and symptoms. The asynchronous interaction pattern further requires clear and effective patient self-description to avoid lengthy conversation, facilitating timely support for patients.

**Method:**

Inspired by the observation that doctors talk to patients with the goal of eliciting information to reduce uncertainty about patients' conditions, we proposed and evaluated a machine learning-based computational model towards this goal. Key components of the model include (1) how a doctor diagnoses (predicts) a disease given natural language description of a patient's conditions, (2) how to measure if the patient's description is incomplete or more information is needed from the patient; and (3) given the patient's current description, what further information is needed to help a doctor reach a diagnosis decision. This model makes it possible for an online consultation system to immediately prompt a patient to provide more information if it senses that the current description is insufficient.

**Results:**

We evaluated the proposed method by using classification-based metrics (accuracy, macro-averaged F-score, area under the receiver operating characteristics curve, and Matthews correlation coefficient) and an uncertainty-based metric (entropy) on three Chinese online consultation corpora. When there was one consultation round, our method delivered better disease prediction performance than the baseline method (No Prompts) and two heuristic methods (Uncertainty-based Prompts and Certainty-based Prompts).

**Conclusion:**

The disease prediction performance correlated with uncertainty of patients’ self-described symptoms and conditions. However, heuristic solutions ignored the context to decrease large amounts of uncertainty, which did not improve the prediction performance. By elaborate design, a machine-learning algorithm can learn the inner connection between a patient’s self-description and the specific information doctors need from doctor-patient conversations to provide prompts, which can enrich the information in patient self-description for a better performance in disease prediction, thereby achieving online consultation with fewer rounds of doctor-patient conversation.

## Background

Advances in information technologies have boosted the development and adoption of online consultation for health care. For example, haodf.com, the leading online consultation platform in China, has provided service for more than 58 million patients as of 2019 [1]. Supplementing traditional face-to-face consultation, this new channel of patient-doctor interaction has been playing an increasingly crucial role in modern health care systems for several reasons.

First, online consultation can alleviate the problem of imbalanced distribution of precious health care resources. Most medical centers are located in developed areas, which makes it difficult for many people living in rural areas to access high-quality health care services [2]. Online consultation can eliminate the physical distance between doctors and patients, allowing sick people to acquire timely diagnoses from doctors even thousands of miles away from their home.

Second, online consultation helps decrease the load of the health care system. In populous countries such as China, hospitals are often over-crowded with patients. This phenomenon is partly caused by many people going to the hospital for periodic checkups of chronic diseases or preliminary diagnoses, whose health care needs are not critical or urgent. Internet-based diagnoses can triage these patients by helping them decide whether they really need to go see a doctor in person or not. This can help reduce the over-crowding of hospitals and improve the throughput of the health care system.

Third, the unexpected COVID-19 pandemic caused by the severe acute respiratory coronavirus 2 (SARS-CoV-2) in 2019 has attracted ever-increasing attention to contact-free diagnosis methods. As a result, the demand for online consultation sharply increased. According to a report from Xinhuanet, an official news website in China, the number of people consulting doctors through the Internet was more than 100,000 per day across China during the lockdown period, increasing by a factor of 6–7 compared to normal times.^1^

Indeed, online patient consultation has the advantage that patients can conveniently visit doctors almost anywhere anytime through the Internet. Its primary drawback, however, is communication inefficiency [3]. To better understand this problem, let us first recall the process of asynchronous consultation.

Normally, a doctor’s diagnosis starts with the clinician asking a patient what is wrong and the patient giving the doctor a self-description about how he or she feels. Then the doctor continues to ask more questions, such as “Does this part or that part hurt?”, “What medicine did you take?”, ''What is your body temperature?'', and so on. The patient answers these questions, and the doctor may follow up with more questions. This back-and-forth exchange does not end until the doctor reaches a confident diagnosis, or assessment, of the patient's medical problems.

How many rounds of conversation this process requires largely depends on the completeness of the information provided by the patient. In the example above, the patient needed at least four rounds to make the doctor understand his condition. However, if a patient tells the doctor how they feel, which part(s) of his body hurt, and what medicine he has taken in the first round of self-description, the patient will greatly reduce the number of rounds of question-answering with the doctor before a diagnosis is made.

In face-to-face diagnoses, the difference between four rounds and one round of conversation does not matter because any question can be answered almost immediately. However, when the diagnosis is done through the internet, the difference is critical. For example, in online diagnosis in haodf.com, these consultations are not real-time but work like an asynchronous chat. The time it takes to receive a doctor's reply depends on how busy the doctor is with duties offline. Similarly, the patient may not give an instant response for various reasons. Under this situation, a four-round conversation can take much longer than a one-round one. The problem of prolonged consultations may affect user experience extensively for both doctors and patients [4]. Such effects can be magnified in a society like China with a low doctor-to-patient ratio.

To reduce the conversation rounds that doctors need in an online consultation, we aim to seek an approach that can automatically prompt patients to provide more information during their first round of input in an online consultation so that they provide as complete information as possible early in the conversation.

Some studies have directly used powerful machine-learning models to assign disease labels to patients according to their health records [5–11]. This kind of approach assumes that a patient's full information is already collected, which is not the case in online consultation scenarios. Other studies resorted to dialogue systems to automatically guide patients to detail their conditions, and then generate diagnosis results [4, 12–14]. These methods require not only nuanced understanding of patient's language and fluent generation of doctor's language, but also a large and diverse collection of labeled conversation data, which are extremely difficult to create. Other studies select existing answers given a patient's utterance to drive the dialogue [15–17]. The difficulty with such methods is that a large pool of question–answer pairs is required, which is also difficult to obtain.

Building on these studies, we are motivated by the characteristics observed in health care consultations: (1) To measure certainty, doctors need a certain amount of information; (2) doctor-patient conversations involve multiple-choice questions in many cases; (3) doctors converse with patients in order to decrease their uncertainty and increase their confidence about the correct diagnosis. We designed a machine learning based framework to fulfill our aim.

We first trained a disease diagnosis function with full information consisting of patient self-descriptions and doctor-patient conversations in order to measure the certainty for an unseen patient's self-described symptoms; then, we built a collection of potential prompts by selecting top *k* TFIDF words from doctor-patient conversations; finally, we used the prompt and patient self-description pairs to train an information elicitation function, wherein the prompt can increase the prediction of the correct diagnosis. When a self-description is evaluated as under-informative by the diagnosis function, the elicitation function launches to provide prompts to help make it more informative.

In this study, our main contribution can be summarized as follows:

- Designed a machine learning based framework to reduce the rounds of doctor-patient conversation in online consultations.
- Instantiated the proposed framework with different models and prompt strategies.
- Conducted a number of experiments on the different instantiations on three Chinese online diagnosis datasets, and found that the instantiation BERT + Learned Prompts delivered the best performance in most time.

### Prior work

#### Dialogue diagnosis

There are some works studying dialogue between doctors and patients during the medical consultation for diagnosis. Tang et al. [4] proposed a framework that casts dialogue diagnosis as Markov Decision Process and trains the dialogue policy via reinforcement learning. In general, the working process of the framework is like MYCIN [18], it starts with a patient's self-report and inquires symptoms from the patient, this loop will not end until the system meets ending condition. Wei et al. [12] used a similar schema, but adopt deep Q-network to parameterize policy. The two works mostly rely on data-driven learning. To utilize external information, Lin et al. [13] proposed an end-to-end knowledge-based dialogue system to incorporate knowledge graph into dialogue management, and Xu et al. [14] used a symptom graph to implement goal attention mechanism capturing more symptoms related information from dialogue. Unlike all of these works, which utilize the conversation form to do diagnose, our work is to learn from conversation.

#### Answer selection

Guiding user to complete information can be done through providing the answer of most related questions in a question–answer pool. Technically this is an answer selection task. Feng et al. [15] designed six different architectures based on convolutional neural networks (CNN) to select the right answer for a question in insurance domain. In that work, CNN is used to extract the representation of question and answer in text at different steps in proposed framework. Another work also adopts CNN as the sentence presentation extractor [16]. The difference of this work with previous one is it used a non-linear tensor layer at the final layer to compare the similarity of question and answer. The other popular deep learning model—long short-term memory (LSTM) is also applied to this task. Tan et al. [17] designed a bidirectional-LSTM (BiLSTM) based model as baseline and further extend it through mixing a CNN on top of BiLSTM. The difference between our method and the above ones is that we do not find the answer directly instead a kind of hint.

#### Disease diagnosis with machine learning

Machine learning technology has been widely applied in disease diagnosis. Garg et al. [5] applied several feature selection methods and machine learning algorithms on text-based electronic health records to classify ischemic stroke. Malik et al. [7] developed a general framework for recording diagnostic data and used machine learning algorithms to analyze patient data based on multiple features and clinical observations for eye disease classification. Lucas et al. [8] used support vector machine to search patterns in electroencephalography epochs to differentiate patients with Alzheimer disease. Li et al. [6] used existing knowledge base as additional information source to improve rare disease classification performance. These methods only tried to improve the performance making classifier have better generalization ability or incorporating external knowledge, in this study we introduce a more human-like way to reach better performance.

## Method

### Problem formulation

At a high level, prompting patient to provide more complete information can be viewed as an active information-seeking problem [19]. To motivate our problem formulation, we describe a simplified example of diagnosis as follows.

We assume the doctor can differentiate two diseases: pneumonia and enteritis. To reach the diagnosis of pneumonia, a patient has to simultaneously present the following conditions: fever, asthenia, and dry cough. To reach the diagnosis of enteritis, the conditions include fever, asthenia, and diarrhea. If a patient comes to the doctor for consultation and says he has fever and feels asthenic. In this case, according to the above diagnostic rules, the doctor can not determine which of the two diseases the patient has—both are equally possible. To be certain about the diagnosis, the doctor needs to ask whether the patient experienced dry cough or diarrhea. When the third condition is confirmed, the doctor can reach a conclusion: if the patient has dry cough, he probably has pneumonia; otherwise, he is more likely to have enteritis (assuming that dry cough and diarrhea are mutually exclusive).

We make several observations from this example. First, when the information is incomplete, the doctor asks questions to elicit more information. Second, such questions are asked to decrease uncertainty in diagnosing a disease. Third, each question expects a categorical answer. After obtaining further information, the doctor incorporates it with the initial information towards making a diagnosis with more certainty. We now formulate the consultation process as follows.

#### Consultation process

Given a patient's self-description ${\varvec{x}}$ (represented as a vector of information), a doctor attempts to make a diagnosis by mapping ***x*** to ***y***, where ${\varvec{y}}$ is probability distribution over the set of diseases in question. The doctor's mapping/reasoning process can be represented as a function $f$, i.e., ${\varvec{y}} = f\left( {\varvec{x}} \right)$.The most probable disease $y^{*} \in {\varvec{y}}$ would be chosen as the diagnosed disease. If the patient's self-description ${\varvec{x}}$ is complete, the doctor will confidently assert a diagnosis $y^{*}$ with high certainty. However, if ${\varvec{x}}$ is incomplete, the doctor may be uncertain about the diagnosis. In terms of the disease probability vector ${\varvec{y}}$, $y^{*}$ may not have a high enough probability, or multiple diseases may have nearly as high probabilities as $y^{*}$. To reduce uncertainty about the diagnosis, the doctor will need more information ${\varvec{z}}$ to be collected. ${\varvec{z}}$ is another vector of information that answers doctor's follow-up questions after seeing ${\varvec{x}}$. After obtaining ${\varvec{z}}$, the doctor will make a diagnosis again by invoking $\user2{y^{\prime}} = f\left( {{\varvec{x}} + {\varvec{z}}} \right)$. The hope is that this time, the candidate diagnosis $y^{*} \in \user2{y^{\prime}}$ is correctly identified as the most probable disease with high certainty.

#### Contextual prompts

Ideally, a computer algorithm can capture the doctor's follow-up questioning process as a model $g$ that generates the questions ${\varvec{z}}$ based on ${\varvec{x}}$, i.e., ${\varvec{z}} = g\left( {\varvec{x}} \right)$. This allows the online platform to ask follow-up questions as soon as the user typed in his initial descriptions, instead of waiting for the doctor to ask such questions. We call ${\varvec{z}} = g\left( {\varvec{x}} \right)$ *contextual prompts* as the prompts ${\varvec{z}}$ shall depend on the context ${\varvec{x}}$. Such contextual prompts can be useful as it can save doctors and patients from time-consuming asynchronous communications. Instead, the online platform can prompt the patient to enter more information based on what has been entered so far.

Computational modeling of the consultation process with contextual prompts.

The above conceptual formulation includes a few components, which we further instantiate below.

- **Patient's initial self-description** ${\varvec{x}}^{\left( i \right)}$. This is a short natural language document written by the $i$-th patient when they initiate the request for online consultation. Here we assume different documents are written by different patients.
- **Ground-truth diagnosis result** ${\varvec{y}}^{\left( i \right)}$. This is the actual diagnosis given by the doctor to the $i$-th patient. Formally, if there are *m* diseases, then ${\varvec{y}}^{\left( i \right)}$ is a *m*-dimensional one-hot vector with a 1 at the dimension corresponding to the diagnosed disease, and 0 elsewhere.
- **Diagnosis function** $f$. The diagnosis function $f$ takes a patient's self-description (with or without prompts) as input and then outputs a probability distribution ${\varvec{y}}$ over the set of *m* predefined diseases. This function is instantiated as a text classification model trained on the online consultation corpus. Further, as a ''simulated doctor'', this function needs to reason like doctors who made the disease prediction after having complete information of the patient. Thus, we train $f$ with complete information where the input document is a concatenation of patients’ initial descriptions and the follow-up doctor-patient conversation.
- **Uncertainty measure and threshold**. The consultation process involves a decision point: if the predicted disease distribution has uncertainty higher than some threshold ${\uptau }$, follow-up questions (or contextual prompts) shall be invoked. Here we use Shannon's information entropy to measure the degree of uncertainty of a probability distribution [20]. Given a predicted disease distribution vector ${\varvec{y}}$, we calculate the entropy $H\left( {\varvec{y}} \right)$ as its uncertainty measure:
  1 $$H\left( {\varvec{y}} \right) = \mathop \sum \limits_{j = 1}^{m} - y_{j} log\left( {y_{j} } \right)$$

where *m* is the number of diseases, $y_{j}$ is the predicted probability for the $j$-th disease.

In pilot study, we also explored margin (absolute difference between the highest and the second highest probabilities) and confidence (absolute difference between the highest probability and 1/*m*) [21] to measure uncertainty. The impact of different uncertainty measures on experimental results was minimal.

We need a threshold ${\uptau }$ to decide whether the uncertainty is high enough to invoke contextual prompts. We set the average entropy value of the training data as the threshold. That is, for every patient's initial self-description ${\varvec{x}}^{\left( i \right)} \user2{ }$ in the training data, we apply $f$ to obtain a predicted probability vector ${\varvec{y}}^{{\varvec{i}}}$, which has an uncertainty measure $H\left( {{\varvec{y}}^{\left( i \right)} } \right)$. The threshold $\tau$ equals the average of $H\left( {{\varvec{y}}^{\left( i \right)} } \right)$ as $i$ exhausts all ${\varvec{x}}^{\left( i \right)}$'s in the training data.

- **Contextual prompt vector** ${\varvec{z}}$. Each dimension in ${\varvec{z}}$ represents whether to prompt the user to describe his experience about a specific medical term. Knowing information about these terms should help the doctor better assess the patient's condition and make a diagnosis. We apply a data-driven approach to construct this vocabulary of prompt terms. Specifically, we take $k$ words with the highest TFIDF weights from doctor-patient conversations in the training corpus. A predicted contextual prompt vector ${\varvec{z}} = \left[ {z_{1} ,z_{2} , \ldots ,z_{k} } \right]$ has $k$ dimensions. The elements can take real values indicating the predicted utility of prompting a user to mention a term in the follow-up conversation. In this work, our focus is the way of information elicitation, so in order to limit the computation complexity, we limit the prompt vocabulary size $k = 100$.
- **Information elicitation function** $g$. As formulated above, the function $g$ generates the contextual prompt ${\varvec{z}}$ given patient's initial description ${\varvec{x}}$. Since each of the $k$ dimensions in ${\varvec{z}}$ represents whether a term should be present or absent, we can view $g$ as a function that has $k$ real-valued outputs, each estimating an importance score for a term given the context ${\varvec{x}}$. Our instantiations of function $g$ are described in the next subsection.
- **Updating operation** $+ .$ Patients use natural language to revise their initial self-description under the guidance of prompts in the real world. Therefore, we assumed that the given prompts would be mentioned in the new description: ${\varvec{x}} + {\varvec{z}}$ simply appends ${\varvec{z}}$ to ${\varvec{x}}$.

### Instantiating information elicitation function ${\varvec{g}}$

According to the way doctors ask questions, with the goal of decreasing their uncertainty about the correct diagnosis based on the patient’s self-description, we designed a strategy named ''Learned Prompts''.

#### Contextual prompts

We train a classification model ${\varvec{z}} = g\left( {\varvec{x}} \right)$ where ${\varvec{z}}$ is an array of $k$ independent probabilities indicating the chance of prompting a term. This effectively translates $g$ into $k$ independent binary classifiers, each predicting the chance of prompting the $l$-th term, $1 \le l \le k$. To construct training data $\left\{ {\left( {{\varvec{x}}^{\left( i \right)} ,t_{l}^{\left( i \right)} } \right)} \right\}$ for the $l$-th binary classifier, the ground truth label $t_{l}^{\left( i \right)}$ for the $i$-th training instance is determined as follows. $t_{l}^{\left( i \right)} = 1$ if adding $z_{l}$ (the $l$-th prompt term) into the initial description ${\varvec{x}}^{\left( i \right)}$ increases the predicted probability of the diagnosed disease; $t_{l}^{\left( i \right)} = 0$ otherwise. The rationale here is that a term $z_{l}$ should have high chance to be prompted if mentioning it in later conversations would increase the doctor's certainty on the disease that is ultimately diagnosed.

Formally, $t_{l}^{\left( i \right)} = 1$ if ${\varvec{y}}^{\left( i \right)} ,f\left( {{\varvec{x}}^{\left( i \right)} + z_{l} } \right) > {\varvec{y}}^{\left( i \right)} ,f\left( {{\varvec{x}}^{\left( i \right)} } \right)$, where $\langle {\varvec{a}},{\varvec{b}}\rangle$ is the dot product of ${\varvec{a}}$ and ${\varvec{b}}$; ${\varvec{x}}^{\left( i \right)} + z_{l}$ concatenates the document ${\varvec{x}}$ and the word $z_{l}$.

For comparison purposes, we also present three baseline strategies as follows.

#### No prompts

Under this strategy $g$ does not output a prompt. This effectively assumes *no* information elicitation process when making diagnosis.

#### Certainty-based prompts

Given ${\varvec{x}}$, we measure uncertainty for all $f\left( {{\varvec{x}}^{\left( i \right)} + z_{l} } \right)$, $1 \le l \le k$. We then rank prompt terms $z_{l}$ such that the terms that made the disease prediction more certain (has low entropy) are ranked at the top. The rationale is that if knowing more about a term can increase doctor's certainty about the diagnosis, that term may be useful.

#### Uncertainty-based prompts

This strategy was similar to that of Certainty-based Prompts but ranked prompt terms in reverse order (prioritizing terms that made the prediction more uncertain). This is inspired by the uncertainty-based sampling method in active learning [21].

#### Selecting top prompts

When an online platform prompts a user to say more about their medical conditions, the prompt may only contain a small number of terms (e.g., ''Can you continue to describe aspects *x*, *y*, and *z''*?). Therefore, we only consider the top $q$ terms ranked by their scores as assigned by $g$. In this study, we vary $q$ across the range $\left\{ {1,2,3,4,5,6,7,8,9,10} \right\}$.

**Fig. 1:**
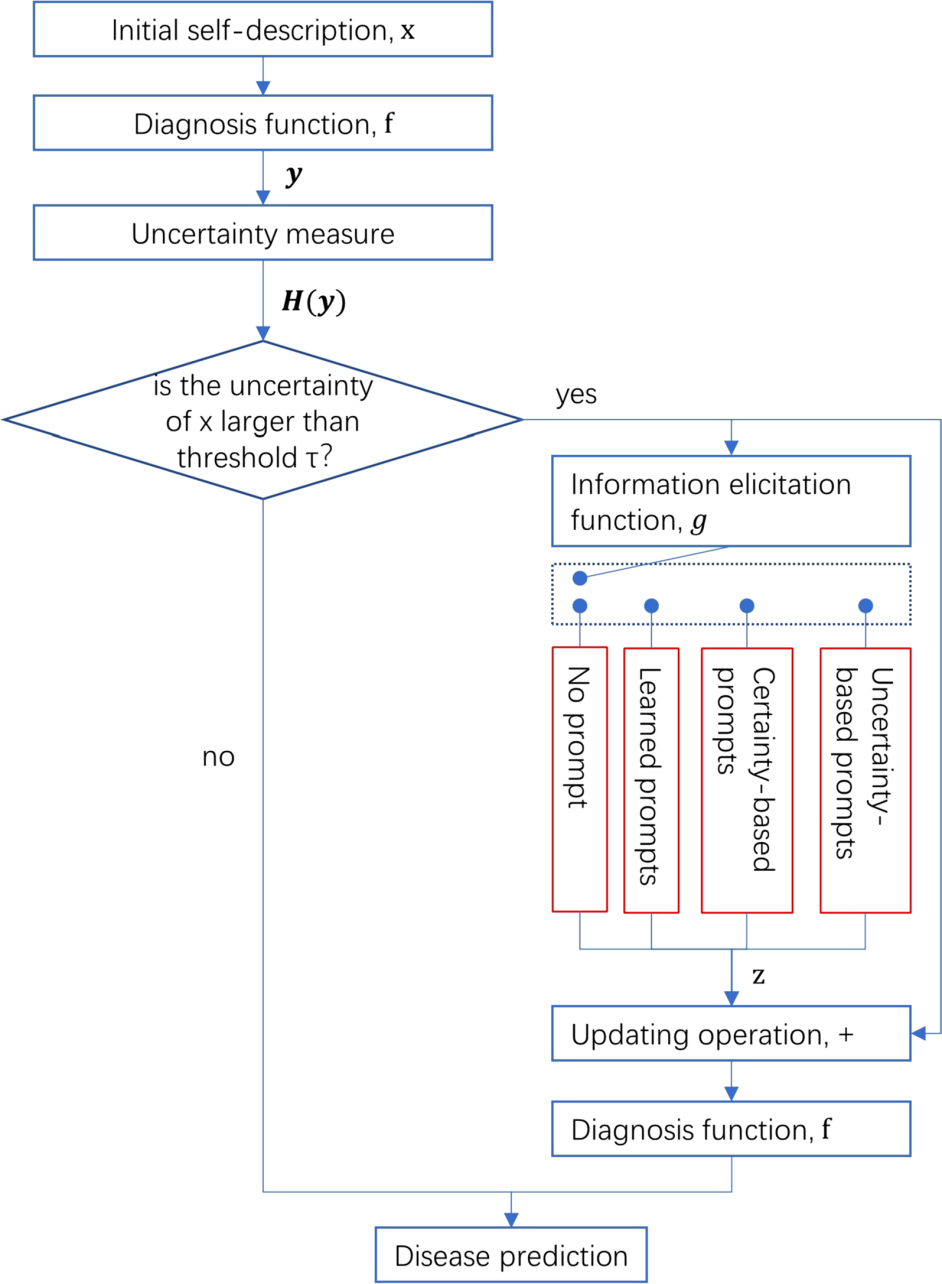
Workflow of diagnosis with prompts

Summarizing the above description, Fig. 1 depicts the overall workflow of the proposed method. The dashed box denotes a switch that controls which strategy the information elicitation function $g$ chooses.

### Compared methods

Because both $f$ and $g$ are classifiers, we explored two kinds of models: The one was a traditional classifier working on sparse representation, like bag-of-words (BOW), that is logistic regression; we use BOW to denote this model in the rest of this paper. The other model was Bidirectional Encoder Representations from Transformers (BERT) [22]. Because the datasets we used below are Chinese, we configure the Chinese BERT-base model released by Google.^2^ Each model can work with four prompts strategies, thus we had eight methods to compare. We named each method with the unified pattern ''model name + prompts strategy''. The eight methods are: BOW + No Prompts, BOW + Learned Prompts, BOW + Certainty-based Prompts, BOW + Uncertainty-based Prompts, BERT + No Prompts, BERT + Learned Prompts, BERT + Certainty-based Prompts, and BERT.  + Uncertainty-based Prompts.

### Experiment and evaluation

#### Data description

**Fig. 2:**
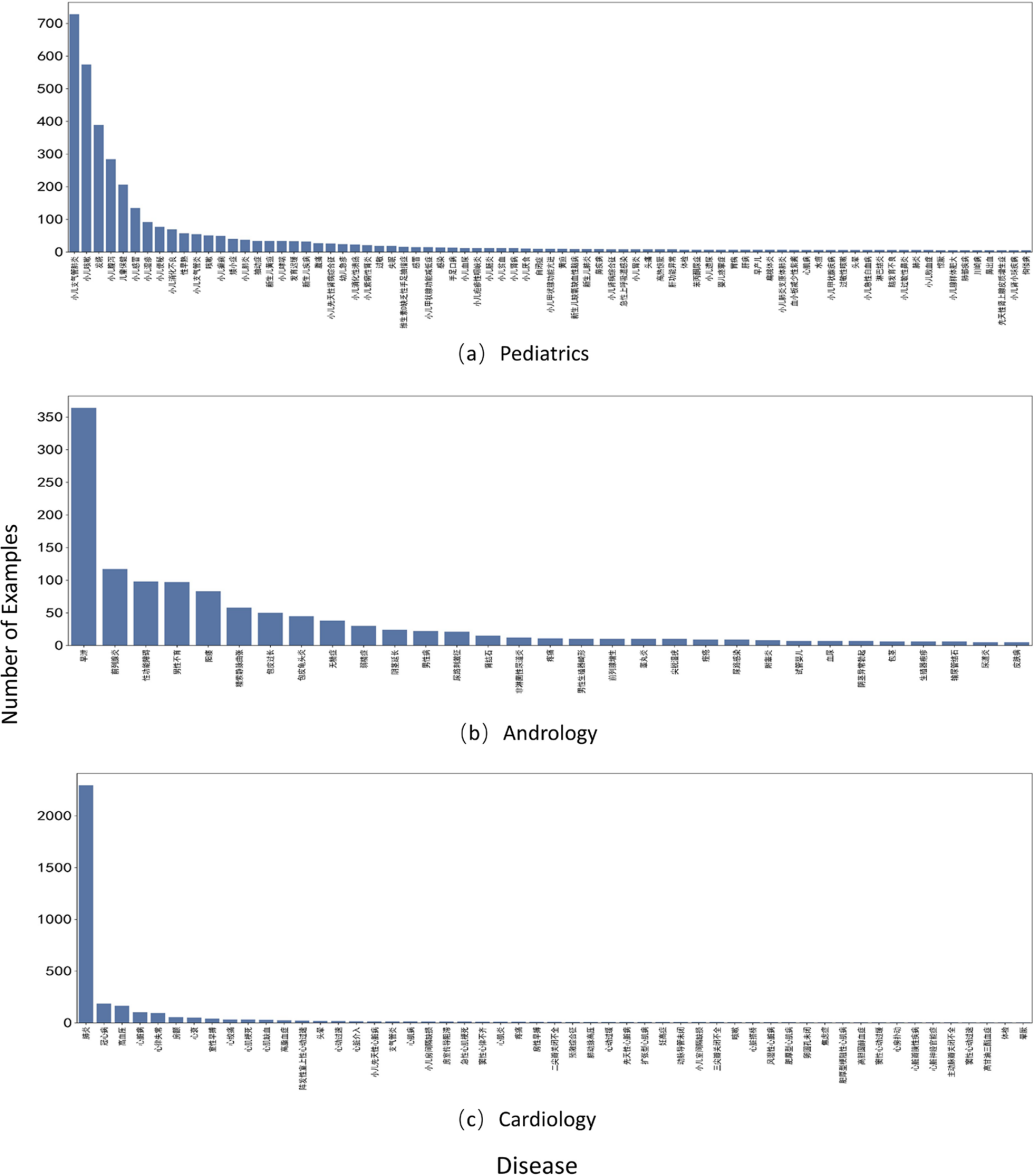
The data distribution of three departments

We used three Chinese patient diagnosis datasets to demonstrate the effectiveness of our method. They were from three different areas of medicine: pediatrics, andrology and cardiology. Figure 2a–c show the distribution of the three datasets, respectively.

**Fig. 3:**
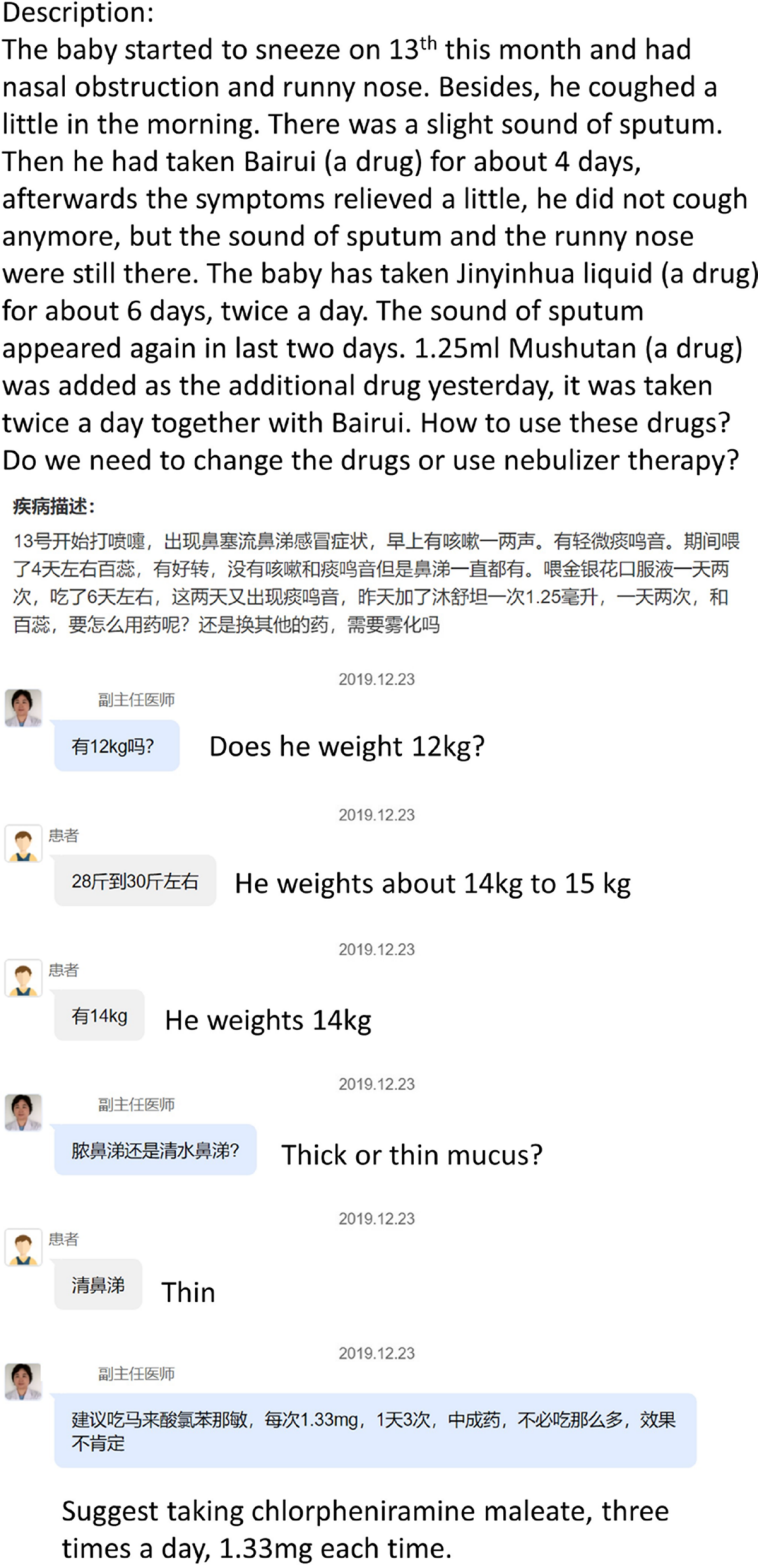
A screenshot of a diagnosis on haodf.com. The doctor's statements are in light-blue bubbles. The patient's statements are in light-gray bubbles. We include English translation of the Chinese post to improve readability. Source: https://www.haodf.com/bingcheng/8821240724.html. Accessed in June 2021

The corpora are all from haodf.com, the largest Chinese online platform that connects patients to doctors. On the platform, a diagnosis starts with a patient's main concerns in text. Then a doctor converses with the patient to give his or her suggestion or ask more questions to better understand the patient's condition. In the end, the doctor uses a disease to label this consultation. We illustrate the data pattern on haodf.com in Fig. 3

Each document consists of two parts: initial description (ID) and clarification. An initial description is a patient's self-description of symptoms used to consult a doctor, and a clarification is the conversation between the patient and the doctor. Following the notation we used in the Problem Formulation section, we use $X$ and $C$ to denote the collection of ID and clarification respectively, such that $X = \left\{ {x_{1} , x_{2} , \ldots ,x_{n} } \right\}$, $x_{i}$ denotes the $i$-th example's ID, $C = \left\{ {c_{1} , c_{2} , \ldots ,c_{n} } \right\}$, $c_{i}$ denotes the $i$-th example's clarification. Both $x_{i}$ and $c_{i}$ are text sequence.

The full information of diagnosis should incorporate both ID and clarification, so we denote it with $X_{comp - info} = \left\{ {x_{1} + c_{1} , x_{2} + c_{2} , \ldots ,x_{n} + c_{n} } \right\}$, where $x_{i} + c_{i}$ denotes putting $i$-th example's ID and clarification together to form one text sequence. When training $f$, $X_{comp - info}$ is used, and for test, $X$ is used.

Table 1 summarizes basic statistics of the three corpora. *pkuseg* package was used for Chinese word segmentation [23].

**Table 1:** Corpora statistics

	Pediatrics	Andrology	Cardiology
# of documents	3593	1200	3487
# of diseases	79	31	53
# of rare diseases	12,081	5,478	9,202
Average # of words/ID	33.6	29.8	21.5

#### Evaluation

All paths in Fig. 1 were designed to predict the disease diagnosis as the final output, thus we used the classification metrics of accuracy, macro-averaged F-score, macro-averaged area under the receiver operating characteristics (ROC) curve, and macro-averaged Matthews correlation coefficient (MCC) to evaluate the performance. To reveal the correlation of prediction and diagnosis uncertainty we also used entropy as a metric.

Viewing the classification of each individual disease as a binary classification problem, results can be divided into true positive (TP), true negative (TN), false positive (FP) and false negative (FN).

Accuracy. Accuracy measures the proportion of right predictions without considering the difference among classes. The metric was calculated as follows:

2 $${\text{accuracy}} = \frac{TP + TN}{{TP + FP + FN + TN}}$$

Macro-averaged F-score. F-score is the harmonic mean of precision and recall, a metric that balances the two [24]. Recall measured the percentage of TPs among all documents that truly mentioned that disease; precision measured the percentage of TPs among all documents predicted to mention that disease. The metric was calculated as follows:

3 $$\begin{array}{*{20}c} {{\text{F}}-{\mathrm{score}} = \frac{2 \times recall \times precision}{{recall + precision}} = \frac{2 \times TP}{{2 \times TP + FP + FN}},} \\ \end{array}$$

To measure the classification performance of a set of diseases, we used the macro-averaged F-score. Formally, the metric was calculated as follows:

4 $$\begin{array}{*{20}c} {{\text{macro-averaged F-score}} = \frac{1}{\left| C \right|}\mathop \sum \limits_{i = 1}^{\left| C \right|} {\text{F-score}}_{{\text{i}}} ,} \\ \end{array}$$

where $C$ is the set of diseases (classes), and ${\text{F-score}}_{{\text{i}}}$ is the ${\text{F-score}}$ of the $i$-th disease.

Macro-averaged Area Under the ROC Curve (Macro-averaged AUC). The ROC curve shows the performance of a classification model at all classification thresholds. The curve plots two parameters: true-positive rate (TPR) and false-positive rate (FPR). The two parameters are defined as follows:

5 $$\begin{array}{*{20}c} {TPR = \frac{TP}{{TP + FN}},} \\ \end{array}$$

6 $$\begin{array}{*{20}c} {FPR = \frac{FP}{{FP + TN}}} \\ \end{array}$$

Area under the ROC curve (AUC) measures the entire two-dimensional area underneath the entire ROC curve [24]. In practice, the calculation of AUC often adopts the Wilcoxon-Mann–Whitney test [25]:

7 $$\begin{array}{*{20}c} {{\text{AUC}} = \frac{{\mathop \sum \nolimits_{{t_{1} \in D^{0} }} \mathop \sum \nolimits_{{t_{1} \in D^{1} }} 1|f\left( {t_{0} } \right)\left\langle {f\left( {t_{1} } \right)} \right| }}{{\left| {D^{0} } \right| \times \left| {D^{1} } \right|}},} \\ \end{array}$$

where $1|f\left( {t_{0} } \right)\left\langle {f\left( {t_{1} } \right)} \right|$ denotes an indicator function that returns 1 if $f\left( {t_{0} } \right) < f\left( {t_{1} } \right)$ otherwise it returns 0; $D^{0}$ is the set of negative examples, and $D^{1}$ is the set of positive examples. Macro-averaged AUC was used to calculate the average AUC of all diseases:

8 $$\begin{array}{*{20}c} {{\text{macro-averaged AUC}} = \frac{1}{\left| C \right|}\mathop \sum \limits_{i = 1}^{\left| C \right|} AUC_{i} ,} \\ \end{array}$$

where $C$ was the set of diseases(classes) and ${\text{AUC}}_{{\text{i}}}$ was the ${\text{AUC}}$ of the $i -$ th disease.

Macro-averaged Matthews Correlation Coefficient (Macro-averaged MCC). The Matthews correlation coefficient (MCC) calculates the Pearson product–moment correlation coefficient between actual and predicted values [26]. The formula to calculate MCC is as follows:

9 $$\begin{array}{*{20}c} {{\text{MCC}} = \frac{TP \times TN - FP \times FN}{{\sqrt {\left( {TP + FP} \right)\left( {TP + FN} \right)\left( {TN + FP} \right)\left( {TN + FN} \right)} }},} \\ \end{array}$$

Considering the weight of each disease equally, the macro-averaged MCC was calculated as follows:

10 $$\begin{array}{*{20}c} {{\text{macro-averaged MCC}} = \frac{1}{\left| C \right|}\mathop \sum \limits_{i = 1}^{\left| C \right|} MCC_{i} ,} \\ \end{array}$$

where $C$ was the set of diseases(classes) and ${\text{MCC}}_{{\text{i}}}$ was the ${\text{MCC}}$ of the $i$-th disease.

Entropy. Entropy was introduced in Method section. Here we use averaged entropy as the metric:

11 $$\begin{array}{*{20}c} {{\text{averaged}} \;H = \frac{1}{\left| N \right|}\mathop \sum \limits_{i = 1}^{\left| N \right|} H_{i} ,} \\ \end{array}$$

where $N$ is the size of test example in a dataset.

#### Train-test split

To reduce the variance of results caused by the train-test split, we ran a fivefold cross-validation, where 4 folds of the data are used as training and onefold of the data are used as test. The final results are the averaged results of 5 folds. To avoid the case where some classes do not appear in the training or test set, we applied the stratified k-fold.

## Results

**Fig. 4:**
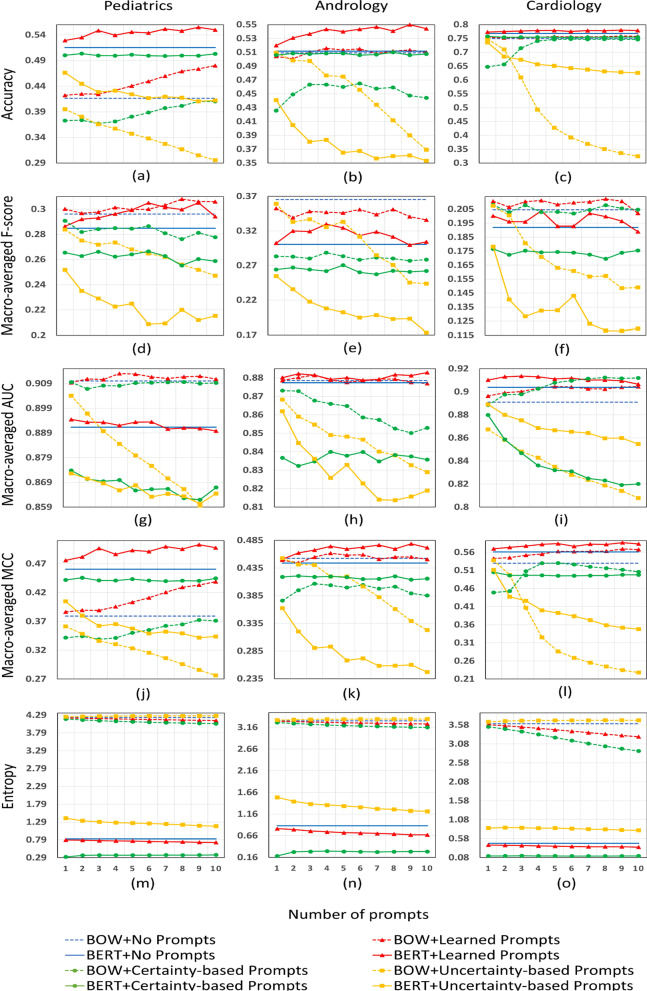
All experimental results on the three corpora are exhibited in this figure. The whole figure consists of 15 subplots, **a**–**o**. Each column of the figure is one data set, each row is one metric. In one subplot, the x-axis is the number of prompts, y-axis is the corresponding metric. For instance, subplot (**a**) summarizes the accuracy of all methods from 1 to 10 prompts on pediatrics data set. The higher the accuracy, macro-averaged F-score, macro-averaged AUC and macro-averaged MCC the better. The lower the entropy, the better

Figure 4 shows the accuracy, macro-averaged F-score, macro-averaged AUC, macro-averaged MCC and entropy of all eight methods under various numbers of prompts on three evaluation corpora. This figure consists of 15 subplots, each one reporting results of one metric of all methods with 1 to 10 prompts on one corpus.

In terms of accuracy (subplots a, b, c in Fig. 4), BERT + No Prompts consistently outperformed BOW + No Prompts across three corpora. When learned prompts were involved, the two baseline methods improved accordingly: BERT + Learned Prompts consistently delivered better performance than BERT + No Prompts, and the performance of BOW + Learned Prompts exceeded that of BOW + No Prompts on two of three data sets. Certainty-based prompts did not help much: both BERT + Certainty-based Prompts and BOW + Certainty-based Prompts performed slightly worse than baseline methods. The two methods related to uncertainty-based prompts performed much worse than baseline.

In terms of macro-averaged metrics, in F-score (subplots d, e, f in Fig. 4), BOW + No Prompts performed better than BERT + No Prompts over all corpora. Apart from andrology, learned prompts benefitted the other two models. But both certainty-based and uncertainty-based prompts almost always hurt the performance of the two models. As for AUC (subplots g, h, i in Fig. 4) and MCC (subplots j, k, l in Fig. 4), there was not a consistent pattern for the baseline methods, but learned prompts consistently improved them across all datasets and helped BERT achieve the best performance on two of three datasets in AUC and all three datasets in MCC. Certainty and uncertainty based strategies still did not help in most cases in the two metrics.

In terms of entropy (subplots m, n, o in Fig. 4), BERT showed much lower entropy than BOW, and there was a similar pattern in entropy over all corpora: BOW + Uncertainty-based Prompts > BOW + No Prompts > BOW + Learned Prompts > BOW + Certainty-based Prompts > BERT + Uncertainty-based Prompts > BERT + No Prompts > BERT + Learned Prompts > BERT + Certainty-based Prompts.

As more prompts are adapted, learned prompts tend to be more helpful, although such benefits are not consistent with the number of prompts.

## Discussion

It is easy to observe one trend: there is a performance gap between two classifiers in disease prediction. From the results delivered by BERT + No Prompts and BOW + No Prompts, we can see that BERT has better performance than BOW in accuracy. This is partly due to the Transformer's multi-head attention mechanism, which allows BERT to learn long-distance dependency efficiently. Another reason is BERT's unique pretraining objective, which can incorporate the sequence information of text in two directions efficiently.

When it comes to macro-averaged metrics, BOW was not always worse than BERT, especially in F-score, where BOW consistently outperformed BERT. This is because BERT has relatively poorer performance than shallow conventional models, such as SVM, on classes with few samples [27]. Each dataset in experiments had nearly 50% classes (diseases) with fewer than 10 examples (eight for training): these included 15 of 31 in andrology, 41 of 79 in pediatrics and 28 of 53 in cardiology. So, the macro-averaged F-score delivered by BERT was lower than that of BOW. In addition, besides the high proportion of minority classes (those with limited examples), data distribution was highly skewed, which made classifiers biased toward predicting major classes (those with more examples) [28], and F-scores of the two classifiers were much lower than their accuracy.

Another intriguing observation is that the trivial solution for decreasing uncertainty did not improve disease prediction. As we described, because of the lack of information in self-description, doctors may be too uncertain to make an accurate diagnosis. So good performance on disease prediction should correspond with low uncertainty. Uncertainty-based and learned prompts did follow the hypothesis: compared to baseline, the uncertainty-based prompts increased the uncertainty while decreasing the performance, the learned prompts decreased uncertainty while increasing performance. But the certainty-based prompts failed to follow this path: searching to quickly lessen large amounts of uncertainty hurt the prediction performance most times. To explore the reason, we use an example (shown in Table 2) from a pediatrics department; this example was classified correctly by BERT + Learned Prompts but incorrectly by BERT + No Prompts, BERT + Certainty-based Prompts and BERT + Uncertainty-based Prompts.

**Table 2:** An example for case study

Initial description:	无感冒症状, 突然发烧, 嗓子红肿, 为何输液又烧?(There are no cold symptoms, got fever suddenly, and throat got inflamed. Why did he fever while receiving transfusion treatment?
True label:	Fever
No Prompts:	None
Predicted label:	Cold
Learned Prompts:	左右 (about), 退烧药 (antipyretics), 血常规 (blood routine examination)
Predicted label:	Fever
Certainty-based Prompts:	咳嗽 (cough), 鼻涕 (runny nose), 病毒 (virus)
Predicted label:	Cough
Uncertainty-based Prompts:	复查 (re-examination), 主任 (director), 体重 (weight)
Predicted label:	Cold

In this example, the self-description is short and involves common symptoms relating to several diseases, like cough, cold and fever. It is unlikely to be classified correctly without additional information. But neither the certainty-based nor the uncertainty-based prompts helped the prediction.

The certainty-based prompts were all related to the cough class. Naturally, these words guided the classifier to be biased toward the cough class; therefore, the predicted probability distribution was more concentrated than in the baseline method, lessening uncertainty. But the certainty-based strategy only considered the decrease of uncertainty and ignored the exactness of prompts, making the classifier like a doctor who is eager to make his decision but lacks comprehensive inquiries. In contrast, the uncertainty-based prompts were too general; those prompts seemed to relate to every disease. They were not helpful to assign correct labels and might have led classifiers to give a more even probability distribution over all diseases than baseline methods, which resulted in the increase in uncertainty. In considering both uncertainty and exactness at the same time, the learned prompts complemented the self-description with related information, thus leading to a correct prediction. In reality, if the patient followed these prompts to complete his initial self-description, the doctor might have had a better chance of getting the correct diagnosis even without further conversation. At the same time, more learned prompts possibly covered the lack of provided information, which therefore resulted in better prediction performance.

However, when self-descriptions only include little diagnosis-related information or are too complex, even Learned Prompts do not work well. We list such two examples, which BERT + Learned Prompts failed to classify, in Table 3. This is a reasonable phenomenon. For the under-informative cases, even doctors need to ask questions from the start to get clues for diagnosis, so it is understandable that Learned Prompts did not know what to suggest and failed to give the right information in such cases. And for complicated cases, the intuitive but trivial strategy was not capable of capturing the key points to give effective suggestions.

**Table 3:** Case misclassified by BERT + learned prompts

Initial description:	Initial description:
医生你好, 我女儿今天早上起来后不舒服?(Hello doctor, my daughter complained of feeling not well when she got up in the morning.) Doctor–patient conversation: Doctor: 宝宝体温是多少?(What is the body temperature of the baby?) Patient: 37.8 (37.8 Celsius) Patient: 她今天比平时吃得少. (She ate less than usual today.) Doctor: 排便是否正常? (Is her defecation normal?) Patient: 早上拉了稀。(She had diarrhea this morning.) Doctor: 如果最近没有接触过肺炎患者, 有可能是发烧。(If she did not contact COVID-19 carrier, she may get fever.) True label: Fever	宝宝前面加米粉又加了胡萝卜泥就开始拉。便便是水和泡沫。一天最多拉到8次化验了大便也没有异常。吃了思密达和金双歧大概有10天左右的时间, 现在是拉大便依然有那种鼻涕状和长丝一天也有5 ~ 6次昨天发烧了感冒吃退烧药布洛芬和抗感颗粒。(My baby started to have loose bowels after eating rice flour and grated carrots. Her stool is watery and foamy. And she even had loose bowels 8 times one day. Stool examination does not show abnormality. She has taken Smecta and Golden Bifid for about 10 days, but her stool is still filamentous like snot. Besides she got fever yesterday and had taken Ibuprofen and Anti-Cold Granule.) (Doctor-patient conversation is omitted) True label: Infantile Diarrhea
Learned Prompts: 睡觉 (sleep), 检查 (examination),时间 (time)	Learned Prompts: 复查 (re-examination), 头孢 (cephalosporin), 鼻涕 (runny nose)
Predicted label: Dyspepsia in children	Predicted label: Cold

In general, Learned Prompts can bring improvement, but it worked relatively poorly with BOW on the andrology corpus. This is related to the characteristics of the department. Andrology has more across-disease keywords than the other two departments: 22.22 in andrology, 19.83 in cardiology and 18.5 in pediatrics. Adding prompts consisting of those words directly might blur the difference among classes for the traditional classifier, which applies a bag-of-words model to represent examples [29], in contrast BERT, which benefits from the self-attention mechanism, which can better capture the slight differences than BOW can. Therefore, the learned prompts hurt BOW but still benefit BERT.

### Limitations

There are some limitations in this study: (1) The way patients were cued to provide further information and (2) the way elicited information was incorporated. First, the natural way to elicit complementary information is the way doctors do it—by asking questions with understandable and complete sentences; our system's pop-up word prompts are not as user-friendly as a natural conversation and may lead to some patient confusion. Second, when people see prompts, they tend to incorporate the new information by revising their self-description in natural language; however, the current updating operation is relatively primitive and might make the useful information noise by missing the patient's syntax.

## Conclusion

In this paper we introduced a method to deal with a problem in Chinese online health care consultation: low communication efficiency caused by under-informative patients' self-descriptions of the problem. The method consists of several parts, including a diagnosis function, an uncertainty calculation function, a prompts pool and an information elicitation function.

The diagnosis function was implemented with a disease classifier trained using comprehensive information from both doctors and patients; the uncertainty calculation function was implemented with an entropy calculation formula; the prompts pool was constructed using top $k$ TFIDF words from doctor-patient conversations; the information elicitation function adopted a classifier trained with pairs of potential prompts and patient self-descriptions, where the prompt improved the prediction of the description on the right class.

Through experiments conducted on three Chinese online medical consultation corpora, we proved the effectiveness of our method. Although, in general, the better option to implement our method is the powerful pretrained deep learning model BERT, the conventional learning regression model (BOW) also delivered decent results, which were comparable with the BERT baseline method occasionally. This means that when computational resources are limited, our method still works in this task.

In future work, we will conduct more evaluation studies to assess the performance of the method using real-world scenarios.

## Data Availability

The data set analyzed in the current study is available in the following GitHub Repository: https://github.com/bruceli518/Contextual-Prompts.

## Abbreviations

TFIDF: Term frequency-inverse document frequency
BERT: Bidirectional encoder representations from transformers
BOW: Bag-of-words
ID: Initial description

## Footnotes

1 http://www.xinhuanet.com/2020-02/25/c_1125622948.htm

2 https://github.com/google-research/bert
